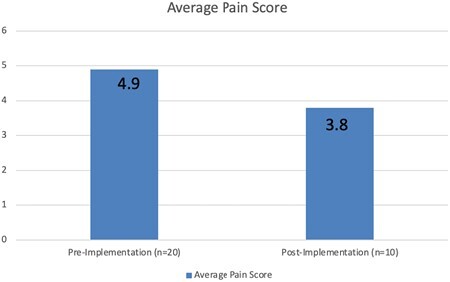# 29 Virtual Reality to Reduce Patients’ Procedural Pain and Anxiety

**DOI:** 10.1093/jbcr/irae036.029

**Published:** 2024-04-17

**Authors:** Hayley Blacker, Mini Thomas, Maria Michelle M Grywalski

**Affiliations:** UCI Health, Irvine, California; UCI Health, Orange, California; UCI Health, Irvine, California; UCI Health, Orange, California; UCI Health, Irvine, California; UCI Health, Orange, California

## Abstract

**Introduction:**

Patients undergo multiple anxiety-inducing and painful procedures when admitted to the hospital setting. Virtual reality (VR) offers an interactive, nurse-driven adjunct to analgesia or anxiolytics. Our burn unit implemented VR as a non-pharmacological opportunity to reduce a patient’s perceived pain and anxiety via dissociative distraction, thereby promoting holistic patient-centered care. After introducing the novel technology to staff, we specifically focused on the potential for virtual reality to improve a patient’s experience during uncomfortable wound care procedures.

**Methods:**

A protocol was developed for the use of VR in the patient care setting with appropriate inclusion/exclusion criteria. Once the protocol was developed, the burn unit nursing staff was in-serviced and encouraged to engage in hands-on practice to increase familiarity. A post-procedure patient survey was developed with two parameters: numeric pain scale and the institution's integrative nursing anxiety scale. Both scales were previously validated and active in the institution’s electronic charting record. This quantitative data was then collected from 20 patients’ individual pain and anxiety perceptions during wound care without VR over the course of one month. Then, the following month, data was collected from 20 patients’ individual pain and anxiety perceptions during wound care with VR implementation. For the VR intervention group, a third parameter was added to the survey that inquired if the patient would recommend VR to other patient’s undergoing wound care.

**Results:**

Patients' average pain score dropped from 4.9 in the control group to 3.8 in the experimental group. Patients' average anxiety score dropped from 2.1 in the control group to 1.8 in the experimental group. These patient-reported survey responses demonstrate that the implementation of VR resulted in a 23% reduction in pain and a 15% reduction in anxiety during wound care procedures. In regards to the third parameter applicable only to the experimental group, 100% of patients reported that they would recommend VR to other patients.

**Conclusions:**

Virtual reality is a nurse-driven, non-invasive modality that reduces a patient’s pain and anxiety during wound care procedures. Furthermore, patients enjoyed their experience with virtual reality as shown in the unanimous response to recommend to other patients.

**Applicability of Research to Practice:**

Virtual reality is a vast realm with exponential opportunities. This low-risk intervention has the potential to optimize a patient’s hospital experience by acting as a modality that reduces patient’s pain and anxiety related to a variety of procedures and therapies led by a multidisciplinary team.